# The knowledge, attitude and practice level of dental auxiliaries regarding oral health care for pregnant patients in the eastern province of Saudi Arabia

**DOI:** 10.12688/f1000research.72903.2

**Published:** 2022-03-28

**Authors:** Daneah Alibrahim, Azza El. Mahalli

**Affiliations:** 1 University Dental Hospital, Imam Abdulrahman Bin Faisal University, Dammam, Saudi Arabia; 2 Department of Public Health, College of Public Health, Imam Abdulrahman Bin Faisal University, Dammam, Saudi Arabia

**Keywords:** Dental auxiliaries; Pregnancy; Oral care; Knowledge; Attitude; Practice; Saudi Arabia

## Abstract

**Background** **:**The purpose of this research was to assess the knowledge, attitude, and practice of dental auxiliaries related to oral health care for pregnant patientsin the Eastern Province of Saudi Arabia.

**Methods: **A cross-sectional study using a questionnaire survey was conducted. The knowledge, attitude, and practice were rated using the Likert scale out of 5. Knowledge and practice were categorized using bloom’s cut off point methods (80% and above isgood, and less than 80% is bad) ***.*** Concerningattitude, (80% and above is positive, and less than 80% is negative) The questionnaires were sent to all  dental hygienists and assistants (N=358), and responses were collected from 218. Statistical Package for the Social Sciences (SPSS) software was utilised to conduct statistical analysis.

**Result** **s** **: **Out of the 358, 218 responded (response rate = 61%). ****More than half of the respondents showed relatively good knowledge (57.3%). Most respondents had a positive attitude (89.4%). Regarding practice, approximately two-thirds had a good practice (67.4%). The knowledge score of hygienists was significantly higher than dental assistants, and respondents with experience in treating pregnant patients had significantly higher knowledge scores than others who did not have experience in treating pregnant patients. There is a statistically significant positive correlation between practice scores and education.

**Conclusion** **s** **: **The findings suggest the need to establish continuous education programs and for dental hygienists and dental assistants to adopt the best practice guidelines on perinatal oral health.

## Introduction

Pregnancy is an experience that most females undergo during their lives, which contributes to physiological and psychological changes. One of the most apparent changes is the one in hormonal levels such as estrogen and progesterone that significantly influences various health issues. Hormonal changes can increase the risk of gingivitis and periodontitis. ^1^^–^^4^ Preventing plaque formation during pregnancy is important for both the mother and the fetus. ^5^ Health care practitioners from diverse backgrounds work together during pregnancy to meet the health and well-being of mothers and their developing fetuses. ^6^ Dental professionals such as dentists and dental auxiliaries (dental hygienists and assistants) are well-positioned to provide oral care for pregnant patients and facilitate referrals to other health care providers. ^3^^,^^6^ To improve the services provided to pregnant patients, researchers need to understand the knowledge and awareness level of dental auxiliaries regarding oral health care for pregnant patients. ^6^ A few studies have been performed in the United States that discuss dental hygienists' awareness, attitude, and practice towards pregnant patients. ^6^ However, in Saudi Arabia, no studies have been conducted to assess the awareness, attitude, and practice of dental auxiliaries towards pregnant patients. Health planners and providers may be better informed from the findings of this study for the benefit of the patients and the public. Dental hygienists are well positioned to provide oral care, introduce pregnancy oral health information, and encourage referrals to other health care providers. Thus, the study aims to assess the level of knowledge, attitude, and practice of dental auxiliaries regarding oral health care for pregnant patients in the Eastern Province of Saudi Arabia.

## Methods

### Ethics statement

Ethical approval was obtained from Imam Abdulrahman Bin Faisal University research ethical review board (IRP-PGS-2020-03-378). Written informed consent was obtained from all subjects who agreed to participate in the study. The confidentiality and privacy of the subjects were maintained.

### Study design and setting

This population-based cross-sectional study was conducted at the governmental dental hospitals in the Eastern province of Saudi Arabia from 17 February 2021 to 17 March 2021.

### Participants

Study participants were all dental auxiliaries (dental hygienist and dental assistant) at the governmental dental hospitals in the Eastern province of Saudi Arabia.

### Data collection and instrument

The ***questionnaire was*** used by Schramm et al. (2016) but modified in the current study. ^6^ It comprised of four sections which included socio-demographic characteristics. Knowledge, attitude, and practice were rated using the Likert scale: 1 = strongly disagree, 2 = disagree, 3 = neutral, 4 = agree, 5 = strongly agree. Knowledge and practice were categorized using Bloom’s cut off point methods (≥80% is good, and <80% is bad). Concerning attitude, (≥80% is positive, and <80% is negative). ^7^ The survey was administered via Google forms services to all dental auxiliaries through e-mail obtained from hospital database. There was no missing data. See the *Extended data* for a copy of the questionnaire.

### Pilot study

A pilot study was conducted on a representative sample of dental auxiliaries from a private hospital. The questionnaire was validated for face and content validity, reliability, and ease of use. Cronbach’s alpha results of the pilot study were acceptable for knowledge (α = 0.778), and attitude (α = 0.791) and good for practice (α = 0.862).

### Analysis

Statistical analysis was performed using SPSS version 23 for Mac. Frequencies and percentages were calculated for categorical variables. The mean and standard deviation were calculated for continuous variables. The median score of 4 was a cut-off point that was considered satisfactory for knowledge, attitude, and practice. ^8^ Mann Whitney and Kruskal Wallis tests were performed to compare the knowledge, attitude, and practice scores. The Spearman rho test was used to correlate the knowledge, attitude, and practice scores with age, education, and experience. A p-value of ≤0.05 was considered statistically significant.

## Results

Of the total of 358, 218 responded, resulting in a response rate of 61% which is considered excellent in most circumstances. ^9^

Table 1 shows the socio-demographic characteristics of study participants.

**Table 1.** Distribution of study participants according to socio-demographic characteristics (2021).

Socio-demographic characteristics	No %
**Age category**	X ± SD (32.2 ± 9.3)
20-	145 (66.5)
35-	67 (30.7)
45-	6 (2.8)
**Gender**	
Male	50 (22.9)
Female	168 (77.1)
**Nationality**	
1-Saudi	152 (69.7)
2-Non-Saudi	66 (30.3)
**Marital status**	
1-Single	107 (49.1)
2-Married	103 (47.2)
3-Divorced	8 (3.7)
**Education level**	
1-High diploma	56 (25.7)
2-Bachelors	155 (71.1)
3-Master (MA, MSc, etc.)	7 (3.2)
**Specialty**	
1-Hygienist	73 (33.5)
2-Dental assistant	145 (66.5)
**Workplace**	
1-Imam Abdulrahman Bin Faisal University	90 (41.3)
2-Dammam medical complex	39 (17.9)
3-Qatif Central Hospital	16 (7.3)
4-King Fahad military complex	55 (25.2)
5-Airbase Hospital	18 (8.3)
**Years of experience**	
1-<5 years	111 (50.9)
2-5-10 years	55 (25.2)
3->10 years	52 (23.9)
**Experience in treating pregnant women**	
1-Yes	138 (63.3)
2-No	80 (36.7)

Table 2 shows that more than half of the study participants (57.3%) had good knowledge regarding oral health care for pregnant patients. Concerning the attitude of dental practitioners towards the pregnant patients, (89.4%) had a positive attitude. Finally, approximately two-thirds of participants had good practice (67.4%).

**Table 2.** The knowledge, attitude, and practice level of dental auxiliaries regarding oral health care for pregnant patients in the Eastern Province of Saudi Arabia (2021).

Variable	No %
**Knowledge**	
1-Good knowledge	125 (57.3)
2-Poor knowledge	93 (42.7)
**Attitude**	
1-Positive attitude	195 (89.4)
2-Negative attitude	23 (10.6)
**Practice**	
1-Good practice	147 (67.4)
2-Bad practice	71 (32.6)

Table 3 shows that the knowledge score of hygienists was higher than dental assistants and of those experienced in treating pregnant women was higher than participants without experience and these differences were significant (P < 0.05).

**Table 3.** Association of knowledge with socio-demographics of dental auxiliaries (2021).

Socio-demographic characteristics	Median	P-value
**Gender**		
Male	4	0.248
Female	4	
**Nationality**		
1-Saudi	4	0.399
2-Non-Saudi	4	
**Marital status**		
1-Single	4	0.359
2-Married	4	
3-Divorced	4	
**Specialty**		
1-Hygienist	4	0.006 *
2-Dental assistant	3.5	
**Workplace**		
1-Imam Abdulrahman Bin Faisal University	4	
2-Dammam medical complex	4	0.333
3-Qatif Central Hospital	3.7	
4-King Fahad military complex	4	
5-Airbase Hospital	4	
**Experience in treating pregnant women**		
1-Yes	4	0.008 *
2-No	3.5	

* P ≤ 0.05.

Table 4 shows that there is no statistically significant difference in the attitude score by gender, nationality, marital status, specialty, workplace nor experience in treating pregnant females (P > 0.05).

**Table 4.** Association of attitude with socio-demographics of dental auxiliaries (2021).

Socio-demographic characteristics	Median	P-value
**Gender**		
Male	4	0.632
Female	4	
**Nationality**		
1-Saudi	4	0.523
2-Non-Saudi	4	
**Marital status**		
1-Single	4	0.134
2-Married	4	
3-Divorced	4	
**Specialty**		
1-Hygienist	4	0.902
2-Dental assistant	4	
**Workplace**		
1-Imam Abdulrahman Bin Faisal University	4	
2-Dammam medical complex	4	0.298
3-Qatif Central Hospital	4	
4-King Fahad military complex	4	
5-Airbase Hospital	4	
**Experience in treating pregnant**		
1-Yes	4	0.486
2-No	4	

Table 5 shows that there is no statistically significant difference in the practice score by gender, nationality, marital status, specialty, nor workplace (P > 0.05). However, there is a significant difference between participants with experience in treating pregnant patients and those without experience (P < 0.05).

**Table 5.** Association of practice with socio-demographics of dental auxiliaries (2021).

Socio-demographic characteristics	Median	P-value
**Gender**		
Male	4	0.935
Female	4	
**Nationality**		
1-Saudi	4	0.629
2-Non-Saudi	4	
**Marital status**		
1-Single	4	0.848
2-Married	4	
3-Divorced	4	
**Specialty**		
1-Hygienist	4	0.168
2-Dental assistant	4	
**Workplace**		
1-Imam Abdulrahman Bin Faisal University	4	
2-Dammam medical complex	4	0.452
3-Qatif Central Hospital	4	
4-King Fahad military complex	4	
5-Airbase Hospital	4	
**Experience in treating pregnant women**		
1-Yes	4	0.009 *
2-No	4	

* Statistically significant at P ≤ 0.05.

Table 6 shows the results of the correlation between knowledge and age, education and experience. There is a positive correlation between the knowledge score and age, education, and experience; however, these correlations are not statistically significant.

**Table 6.** Correlation between the knowledge and socio-demographics of dental auxiliaries (2021).

Socio-demographics	Rho	P-value
Age	0.103	0.128
Education	0.131	0.054
Experience	0.110	0.104

Table 7 shows the results of the correlation between attitude and age, education and experience. There is a negative correlation between the attitude score and age, education, and experience; however, these correlations are not statistically significant.

**Table 7.** Correlation between the attitude and socio-demographics of dental auxiliaries (2021).

Socio-demographics	Rho	P-value
Age	−0.037	0.587
Education	−0.086	0.207
Experience	−0.022	0.742

Table 8 shows the results of the correlation between practice and (age, education and experience). There is positive correlation between the practice score and age, education and experience. However, the only statistically significant correlation is with education (P < 0.05).

**Table 8.** Correlation between the practice and socio-demographics of dental auxiliaries (2021).

Socio-demographics	Rho	P-value
Age	0.063	0.355
Education	0.160	0.018 *
Experience	0.032	0.643

* P ≤ 0.05.

## Discussion

Pregnant women should receive routine and emergency dental treatment; however, there is limited information about the current practices among dental auxiliaries providing care to pregnant women. ^10^

### Knowledge of dental auxiliaries

In this study, dental auxiliaries demonstrated relatively good knowledge (57.3%) regarding oral care provided throughout pregnancy. In addition, they agreed that women should receive preventive dental care during pregnancy (75.2%), which is consistent with the available literature and studies on the subject. ^11^ Of the dental auxiliaries in this study, only 69.7% advocated for the risk of radiographs during this period. Similar disagreement about the use of radiographs for pregnant women has been identified in other studies. ^11^^,^^12^ The current findings demonstrated that the knowledge score of hygienists was significantly higher than dental assistants (p = 0.006). This finding is consistent with a study conducted in North Carolina in 2008 where respondents with low or moderate levels of knowledge were significantly less likely to provide comprehensive treatment for pregnant women than those with high levels of knowledge. ^13^

### Attitude of dental auxiliaries

Dental auxiliaries had a very positive attitude toward perinatal oral health (89.4%). Other studies also found almost universal agreement that dental treatment should be included in prenatal care, with most dentists and dental auxiliaries having favorable attitudes toward pregnancy-specific counselling. ^6^^,^^11^^-^^13^ The present findings revealed that 81.6% of participants believe that they need educational material for pregnant patients. These findings were consistent with the literature where dental auxiliaries required continuing education to improve their awareness about pregnant women’s oral health. ^6^^,^^11^^,^^14^

### Practice of dental auxiliaries

The results of this study revealed that only 36.3% of study participants practiced administration of anesthetic injections. This contradicted previous study results where benzocaine, procaine and lidocaine were administered safely. ^12^^,^^15^ In addition, the current study showed that there was a statistically significant correlation between practice and level of education. This finding has been supported by another study which agreed that continuous dental education could be a useful strategy in improving dental practice. ^12^

### Limitations

In this study, only 218 dental auxiliaries participated and thus the results may not be representative of the dental auxiliaries in the kingdom, and the findings may be not generalizable and may lack validity.

## Conclusion

Dental auxiliaries had positive knowledge, attitudes, and practices regarding offering oral health care to pregnant women. The research results confirmed that many dental auxiliaries shared the desire for continuing education on oral health care during pregnancy to improve oral health practices. Preventive measures for pregnant women would be beneficial not only for the mothers but also for their babies.

## Data availability

### Underlying data

Figshare: main study 2.sav, https://doi.org/10.6084/m9.figshare.17197283.v2. ^16^

This project contains the following underlying data:

- Main study 2.sav (Data for gender, age, nationality, marital status, education, specialty, work place, experience in treating pregnant patients, and questions 11 to 36)

### Extended data

Figshare: main study 2.sav, https://doi.org/10.6084/m9.figshare.17197283.v2. ^16^

This project contains the following extended data:

- Survey for magazine.docx (Questionnaire)

Data are available under the terms of the Creative Commons Attribution 4.0 International license (CC-BY 4.0).
