# Supplementary material for: The knowledge, attitude and practice level of dental auxiliaries regarding oral health care for pregnant patients in the eastern province of Saudi Arabia
Source: F1000Res. 2023 Feb 23;11:216. Originally published 2022 Feb 23. [Version 3] doi: 10.12688/f1000research.72903.3 (PMC10061033; doi:10.12688/f1000research.72903.3)
Supplement: Peer review history

# Peer review: Reviewer response for version 3

**DOI:** 10.5256/f1000research.144529.r164427

**Published:** 2023-03-29

**Authors:** Catherine Maybury

**Affiliations:** 1 Center for Health Literacy, University of Maryland School of Public Health, College Park, MD, USA

Approved. I have no further comments.

Is the work clearly and accurately presented and does it cite the current literature?

Partly

If applicable, is the statistical analysis and its interpretation appropriate?

Yes

Are all the source data underlying the results available to ensure full reproducibility?

Yes

Is the study design appropriate and is the work technically sound?

Yes

Are the conclusions drawn adequately supported by the results?

Partly

Are sufficient details of methods and analysis provided to allow replication by others?

Partly

Reviewer Expertise:

Health literacy, oral health literacy, health education

I confirm that I have read this submission and believe that I have an appropriate level of expertise to confirm that it is of an acceptable scientific standard.

# Peer review: Reviewer response for version 2

**DOI:** 10.5256/f1000research.122785.r159891

**Published:** 2023-01-25

**Authors:** Catherine Maybury

**Affiliations:** 1 Center for Health Literacy, University of Maryland School of Public Health, College Park, MD, USA

The authors have taken an important first step in understanding what dental auxiliaries in their country know and practice related to oral health care for pregnant patients. The article is easy to read and concise. 

 I have a few suggestions.

**Introduction **- The authors state that a few studies have been performed in the US that discuss dental hygienists' awareness, attitude and practice towards pregnant patients but they only cite one article. I did a quick search and found the following (Rainchuso, 2013). ^1^ Please look for additional articles. 

**Methods **- This section should be expanded to explain: a) how you adapted the Schramm instrument; b) how many items were in the survey overall; c) describe the measures (what types of items comprise knowledge, attitudes and practice?); d) for the survey, how many times was the survey link sent to the potential respondents? and e) was the data normal or skewed?

**Discussion **- Knowledge is low (57%). Why is this - is it because they do not receive training? Is it because practices do not treat pregnant women? If ONLY slightly more than half of auxiliaries have adequate knowledge this is a problem. One suggestion is to include this in the training as well as continuing education. 

**Limitations **- Why would the findings lack validity? Please explain.

Is the work clearly and accurately presented and does it cite the current literature?

Partly

If applicable, is the statistical analysis and its interpretation appropriate?

Yes

Are all the source data underlying the results available to ensure full reproducibility?

Yes

Is the study design appropriate and is the work technically sound?

Yes

Are the conclusions drawn adequately supported by the results?

Partly

Are sufficient details of methods and analysis provided to allow replication by others?

Partly

Reviewer Expertise:

health literacy, oral health literacy, health education

I confirm that I have read this submission and believe that I have an appropriate level of expertise to confirm that it is of an acceptable scientific standard, however I have significant reservations, as outlined above.

## References

[1] : Improving oral health outcomes from pregnancy through infancy. *J Dent Hyg* .2013;87(6) :330-5 24357561

# Peer review: Reviewer response for version 2

**DOI:** 10.5256/f1000research.122785.r129028

**Published:** 2022-04-04

**Authors:** Mohammad Abdul Baseer

**Affiliations:** 1 Department of Preventive Dentistry, College of Dentistry, Riyadh Elm University, Riyadh, Saudi Arabia

The authors have made improvements to the manuscript.

Is the work clearly and accurately presented and does it cite the current literature?

Partly

If applicable, is the statistical analysis and its interpretation appropriate?

I cannot comment. A qualified statistician is required.

Are all the source data underlying the results available to ensure full reproducibility?

Yes

Is the study design appropriate and is the work technically sound?

Partly

Are the conclusions drawn adequately supported by the results?

Yes

Are sufficient details of methods and analysis provided to allow replication by others?

Partly

Reviewer Expertise:

Dental Public Health, Preventive Dentistry, Clinical Trials.

I confirm that I have read this submission and believe that I have an appropriate level of expertise to confirm that it is of an acceptable scientific standard.

# Response

**Published:** 2022-04-05

**Authors:** Daneah Alibrahim

**Affiliations:** Imam Abdulrahman Bin Faisal University, Saudi Arabia

Thank you so much Dr. Mohammad Abdul Baseer.

# Response

**Published:** 2022-04-09

**Authors:** azza elmahalli

Greetings

 I'm professor Azza El.Mahalli. The 2nd author of this article. I acknowledge that we amended all the queries raised by Dr Abdul Bassir 

 Regards,

# Response

**Published:** 2022-04-13

**Authors:** Daneah Alibrahim

**Affiliations:** Imam Abdulrahman Bin Faisal University, Saudi Arabia

Dear Dr.Mohammed Abdul Baseer,

 I acknowledge that I did all the amendment for all the queries raised by you. Thank you for the comment, I really appreciate it.

# Response

**Published:** 2022-04-13

**Authors:** Azza El.Mahalli

**Affiliations:** Imam Abdulrahman Bin Faisal University, Saudi Arabia

Greetings

 As a second author, I acknowledge that we did the amendments as requested from Dr Mohamed Abdul Basir

 Thanks,

# Peer review: Reviewer response for version 1

**DOI:** 10.5256/f1000research.76514.r124732

**Published:** 2022-02-25

**Authors:** Mohammad Abdul Baseer

**Affiliations:** 1 Department of Preventive Dentistry, College of Dentistry, Riyadh Elm University, Riyadh, Saudi Arabia

The authors have made a good effort in conducting research on the topic that is not fully addressed in KSA, however:

1. Authors should improve the article's English and grammar.
2. Spelling mistakes in the abstract and other areas should be corrected.
3. The introduction should mention the intended benefits of the study.
4. Under the materials and methods section sample size calculations and sampling techniques utilized in the study should be elaborated.
5. How did the authors obtained email addresses of the participants should me mentioned in the study.
6. Descriptive analysis of each item in the questionnaire should be presented.
7. Normality assessment of the data should be indicated before deciding to opt for non-parametric analysis.
8. Results should also include correlation test among knowledge, attitude and practices.
9. The discussion is very brief, authors should elaborate it by including articles published from Saudi Arabia and elsewhere.
10. Authors should include more study limitations. 
11. References should be updated with more than half being published within last 2-3 years.

Is the work clearly and accurately presented and does it cite the current literature?

Partly

If applicable, is the statistical analysis and its interpretation appropriate?

I cannot comment. A qualified statistician is required.

Are all the source data underlying the results available to ensure full reproducibility?

Yes

Is the study design appropriate and is the work technically sound?

Partly

Are the conclusions drawn adequately supported by the results?

Yes

Are sufficient details of methods and analysis provided to allow replication by others?

Partly

Reviewer Expertise:

Dental Public Health, Preventive Dentistry, Clinical Trials.

I confirm that I have read this submission and believe that I have an appropriate level of expertise to confirm that it is of an acceptable scientific standard, however I have significant reservations, as outlined above.

# Response

**Published:** 2022-03-23

**Authors:** Daneah Alibrahim

**Affiliations:** Imam Abdulrahman Bin Faisal University, Saudi Arabia

Dear Dr. Mohammad Abdul Baseer

 Thank you for the comments, I really appreciate it. I have addressed your comments and submitted the new version.

 best regards
